# Economic evaluation of kinetic energy storage systems as key technology of reliable power grids

**DOI:** 10.1371/journal.pone.0311160

**Published:** 2024-10-28

**Authors:** Stephan Düsterhaupt, Martina Černíková, Šárka Hyblerová

**Affiliations:** 1 Department Mechatronic Systems, Institute for Process Technology, Process Automation and Measurement Technology (IPM), Hochschule Zittau/Görlitz – University of Applied Sciences, Zittau, Germany; 2 Department of Finance and Accounting, Faculty of Economics, Technical University of Liberec, Liberec, Czech Republic; Vellore Institute of Technology, INDIA

## Abstract

In recent years, energy-storage systems have become increasingly important, particularly in the context of increasing efforts to mitigate the impacts of climate change associated with the use of conventional energy sources. Renewable energy sources are an environmentally friendly source of energy, but by their very nature, they are not able to supply the required amount of energy in a uniform distribution. This study evaluated the economic efficiency of short-term electrical energy storage technology based on the principle of high-speed flywheel mechanism using vacuum with the help of an innovative approach based on life cycle cost analysis (LCC). The innovative potential of high-speed flywheel energy storage systems (FESS) can be seen in increasing the reliability of the electricity transmission system with the possibility of providing control power to compensate for residual loads caused by volatile renewable power sources and power sinks. Based on the research conducted, the LCC method was selected in this study as the most appropriate method to evaluate the economic efficiency of a high-speed FESS used to compensate for short-term fluctuations in an upgraded electric transmission system. As a result, the adjusted LCC per MWh values were compared with the average intra-hour margin realisable in the Intra-Day OTE Market, while the margin calculation also considered the efficiency of the inertial storage. Under the modelled technical and economic conditions, it was found that a high-speed FESS project that can compensate for short-term fluctuations in the electricity transmission system can be economically efficient in the Czech Republic.

## 1. Introduction

The energy systems of developed countries are undergoing a fundamental transformation towards sustainability requirements, with the dominance of renewables and the decline of fossil fuels. Although renewable energy sources are environmentally friendly, they cannot supply the required amount of energy in an evenly distributed manner. Owing to the volatility of the power source and demand, various energy-storage systems have become increasingly important.

The European Union is making extensive efforts to restructure its energy infrastructure. With respect to the Paris Climate Agreement, nations around the world are driving forward the transformation of their energy industries and industrial sectors away from fossil fuels and towards renewables. The goal is to achieve a climate-gas-neutral energy economy by 2050. Coal phase-out will be binding upon all EU member states by 2038.

The loss of conventional power plant capacities leads to a reduced supply of spinning reserves and qualified primary control power. However, renewable energy sources can only provide these system services to a limited extent. Therefore, industrial-scale energy storage facilities are necessary to stabilise the European power grid. They can compensate for the residual loads by providing positive and negative control powers required for load control within the grid.

One such solution is the use of autonomous cellular energy systems. These regional balancing groups offer electric power according to demand (location-, time-, and quantity-based) from the point of view of sources, to store it and to obtain it from the point of view of sinks.

Currently, battery technology, primarily based on lithium-ion accumulators, is widespread. These storage systems range in size and capacity, from small batteries for households to large batteries for industrial applications. Owing to their relatively widespread use, these applications have reduced costs and are beginning to compete in price with fossil fuel-based technologies. However, the batteries themselves are problematic owing to their composition. Additionally, the complex mining of lithium poses a certain burden and risk to the environment. Currently, Europe lacks economically viable deposits. Thus, lithium, as a key raw material for changing energy policy, also represents a political lever. Therefore, the scientific and research activities of many countries have been directed towards finding alternative energy storage options.

A large potential was observed in short-term storage systems for electrical energy. The focus is on modular kinetic energy storage systems (KERS), which are to be offered to the technology market using a modular system and function-integrated lightweight construction adapted to the requirements of the selected sectors (energy, transportation, passenger transport, offshore, etc.). The aim of this study is to establish an alternative technology to lithium-ion batteries. This is because KERS, manufactured with modern fibre composites, can be seamlessly integrated into European value chains (mechanical engineering, electrical engineering, electronics, and software).

An innovative and environmentally friendly method of providing control power, especially regarding future demands on power grids, is the storage of kinetic energy in high-speed FESS. At present, these projects are not commercially exploited; however, in the future, they present promising potential for solving the energy storage problem for certain purposes. The element of novelty generates a certain degree of uncertainty in these innovative solutions, not only in the technical, but also in the economic field. The technical parameters are based on assumptions as there are no relevant data from the long-term use of the technology. This was also reflected in the economic evaluations of these projects. There are several well-known, well-described, and widely used techniques for evaluating the economic efficiency of investment projects. However, these methods are unsuitable for evaluating environmental technologies. This is because they primarily work with a monetary expression of all aspects of the investment, assume only selected risks, and do not pay much attention to elements of uncertainty or factors that cannot be monetised. Therefore, to evaluate energy storage projects, it is appropriate to use methods that eliminate these shortcomings to some extent. This study evaluated the economic efficiency of short-term electrical energy storage technology based on the principle of a high-speed flywheel mechanism using vacuum, with the help of an innovative procedure based on the life-cycle cost method. This energy-storage principle is the subject of international research within the framework of university partnerships. The calculation of economic efficiency was based on price levels in the Czech Republic. The total capital cost method was used to determine the costs associated with asset ownership. Individual components were expertly valued and supplemented with operating costs. Under certain technical (performance and lifetime of the technology) and price assumptions (current price of the technology and price of energy), the economic efficiency of the technology can be identified in this way. The discussion should examine the appropriateness of the selected evaluation method, its advantages and disadvantages, and compare it with other economic evaluation methods. It is alo important to identify the aspects that influence the relevance of the results, including macroeconomic factors such as inflation trends, government regulation of the energy market, and future fiscal and monetary policies of individual countries. Currently, energy storage systems pose a challenge for researchers in developed countries. There is no one-size-fits-all solution for all applications. The purpose of research and development is to create an energy storage system that is environmentally friendly and, for a specific task, not only provides a technical solution in the long term but also has adequate economic efficiency.

## 2. Literature review

Today, society faces new challenges regarding energy strategies aimed towards sustainability and environmental protection. Worldwide, countries are transforming their energy systems within the context of the Paris Climate Agreement. The aim is to move away from the use of fossil fuels in centralised large-scale power plants, towards the decentralised use of sustainable energy sources such as the sun, wind, or biomass. The transition to renewable energy sources requires new approaches to securing electricity supply. The use of renewable energies depends on their specific characteristics. Owing to the intermittent nature of many of these sources (solar, wind, and tidal), the supply of this energy is unstable. When renewable energy is used, the energy demand may be lower; however, at peak times, the capacity of these energy sources is exceeded. Thus, the energy supply fluctuates (daily, monthly, seasonally and annually), creating a reliability problem [1, 2]. One possible solution to the problems associated with the temporal mismatch of supply and demand of renewable energy sources is the use of energy storage systems.

Currently, energy storage system (ESS) projects are highly desirable in society and are widely discussed [3, 4]. ESS are essential technologies for the modernisation of the electric grid. The balance between supply and demand, stability, control of voltage and frequency delays, and improvement in the quality of electricity supply are all important attributes that make ESS-type technologies suitable for application.

The demand for renewable resources and environmentally friendly storage systems is accelerating research into new materials and technologies to improve the performance and durability of ESS. Their environmental and economic impacts are also being assessed. Different energy storage systems are currently being investigated [5] and a range of possible ESS solutions are being discussed by the scientific community [6]. Different approaches to energy storage raise questions not only in terms of ecology or technical design, but also in terms of the economic efficiency of the selected technologies.

Most renewable energy sources are decentralised. Owing to their lower power density compared to conventional power plants, several converters are combined within virtual power plants. Districts, communities, and individuals are trying to achieve greater self-sufficiency by building local storage solutions. In recent years, this new societal need has led to research activities in storage technologies and an increased demand for stationary batteries that can satisfy energy storage requirements owing to their technical parameters. Battery storage systems can have different sizes and capacities, ranging from small batteries for households to large batteries for industrial applications. The efficiency of battery storage has significantly improved over the years, and the cost of production has decreased [4]. This makes battery storage systems increasingly competitive with traditional energy sources, such as fossil fuels [7]. The biggest advantage of this energy storage technology is its large capacity and power. However, this method also has several disadvantages. The battery lifetime is a few years and decreases with a higher discharge current and higher voltage at which it is charged. It is dependent on the ambient temperature; as the temperature increases, its maximum capacity decreases. Improper use or mechanical damage can result in ignition and explosion hazards. Batteries are relatively expensive to manufacture, and their recycling is difficult and costly.

Current scientific knowledge offers multiple ways to store energy, including electrochemical, electrical, magnetic, mechanical, and thermal systems. The mechanical approach, represented by flywheel energy storage systems (FESS), has been scientifically evaluated as one of the most progressive energy storage methods. The advantages of this system include high performance quality, higher charge and discharge cycle rates, higher efficiency, and longer lifetime. The system can operate for more than 30 years without significant performance degradation. Replacement and repair costs are much lower than those of battery energy storage systems. FESS also require less maintenance than battery-based energy storage systems. It also has the advantage of a relatively low environmental impact compared to battery technologies (it does not contain potentially hazardous chemical components inside the storage); therefore, the disposal costs of FESS are negligible [8].

The main disadvantage of the FESS is its relatively high self-discharge. Therefore, FESS are particularly suitable for electrical network applications that require short-term storage. Examples include power smoothing and peak reduction, in which the energy storage mainly absorbs short-generation peaks or covers short-consumption peaks. These peaks are often associated with the generation of electricity from renewable sources, particularly wind power. Similarly, this system is also suitable for the frequency control area, where energy storage is only used for short periods, and self-discharge is therefore not a major problem. At the same time, the advantages of FESS, such as a virtually unlimited number of cycles and fast response providing very high performance, can be fully exploited [8]. The use of FESS under specific climatic conditions can be problematic (e.g. low temperatures can make them less efficient). In summary, the FESS is a very interesting and innovative technology with great potential for relatively wide applications. It is important to investigate whether this technology is the best choice for a particular practical scenario.

The financial cost of a system is also an important factor in deciding whether to use a particular technology [9]. Although prices are expected to decrease in the future as technology becomes more widely used, other energy storage systems may still be more advantageous for some applications. The decision to use a particular energy storage technology should not only analyse the technical parameters but also reflect the economics of the project.

Economic evaluation of energy storage projects is not straightforward. Environmental projects are difficult to evaluate using traditional models used for conventional investment projects. When evaluating environmental projects, it is necessary to develop evaluation models that reflect a wide range of effects arising from these investments (including non-monetary effects and positive or negative externalities). It is always necessary to work with certain assumptions that respect the uncertain evolution of the prices of certain commodities and assume the evolution of relevant legislation, including environmental regulation by supranational bodies.

The difficulties associated with evaluating the effectiveness of an investment project are evident not only on the expenditure side (it is not always easy to specify the expenditure primarily related to the environmental objectives of the investment project). Similar problems are associated with identifying the benefits arising from the intended investment. All future benefits are uncertain and carry potential risks. When assessing these types of projects, it is necessary to work with the widest possible source of information to identify potential risks and assess their acceptability. Conventional investment appraisal methods can be used in part to make relevant decisions; however, several other components that affect the decision-making process must be expertly assessed. For the complexity of evaluating individual investment projects, it is necessary to accept "soft" and less quantifiable data as a necessary component of information for quality decision-making processes. The problem remains in quantifying these factors in financial terms: estimating their financial value is complicated and sometimes inaccurate [10].

When assessing the effectiveness of ESS, it is necessary to work with an educated guess with a relatively large degree of uncertainty. These evolving technologies are new, therefore, the economic efficiency of these devices is not well-developed in the literature. Cost data are scattered from different times and energy markets, and are calculated or estimated based on different methods. Because most ESS technologies are in the early stages of development and demonstration, their cost data cannot be conveniently scaled to larger or smaller sizes. Determining the cost per unit of performance is also problematic because, for example, storage size may be different for the same unit of performance. Therefore, it is necessary to work with more detailed information and technical data, such as the storage size, expected efficiency, and declared lifetime of the device under consideration [11].

The basic attributes for examining the cost-effectiveness of energy storage systems are the cost of the storage systems (with a certain performance, efficiency, and lifetime) and the operation and maintenance costs. Expert sources offer a fairly broad overview of methods that can be used for the economic evaluation of technologies, such as Rotella et al. [12] and Jülch [11]. While some authors explore the possibility of evaluating ESS projects using methods commonly used for investment decision-making, other authors lean towards methods that better reflect the specificities of environmental projects.

In studies by Li [1] and Van der Stelt [13], the Payback (PB) method is used to assess the cost-effectiveness of an ESS. This is the simplest method for assessing the economic efficiency of projects, and expresses the time required to cover the initial investment with the revenue or income generated by the investment. An investment is acceptable if the PB is shorter than the expected lifetime of the investment. However, the PB has a potentially high degree of inaccuracy because it does not consider the time value of money. Chiacchio [14] and Lorenzi [15] used the Discounted Payback (DPB) method, in which the time value of money is included in the calculation of the economic efficiency of the ESS. Return on Investment (ROI) is also a simple method for evaluating investment projects. The profitability of an investment project is calculated as the ratio of the net profit to the amount invested. This method was used for the economic evaluation of ESS projects by Liang [16] and Mulleriyawage [17]. The Net Present Value (NPV) method, which is based on the concept of discounted cash flow, is mostly used for investment decision-making. In the field of the economic evaluation of ESS, authors such as Martinez-Bolanos [18] and Bai [19] have employed this method. Tsai [20] and Chagnard [21] evaluated ESSs economically using the Internal Rate of Return (IRR) method. Here, too, the present value of the cash flows of the investment is worked with. IRR represents the interest rate, which makes the NPV of all project cash flows equal to zero. An investment is considered economically acceptable if the calculated IRR is higher than the investor’s minimum required return, which can be derived from the market conditions.

The life cycle cost analysis (LCCA) method, sometimes also referred to as net present cost (NPC), is often used to evaluate environmental investments. The method is based on the NPV and provides the present value of the investment costs and recurrent operating costs incurred over the entire lifetime of the ESS. These costs are presented on an annual basis or per charge or discharge cycle. To assess the acceptability of an investment, costs must be compared with the potential revenue of the investment. LCCA is used, for example, in studies by Jumare [22], Zakeri [23] and Shabani [24].

The Levelised Cost of Storage (LCOS) method has been proposed to compare the cost of electricity generation from renewable and conventional sources. In the case of ESS, it is applied as a Levelized Cost of Electricity (LCOE) [25]. The efficiency indicator (called the economic performance index of the system) is generally calculated as the ratio between the sum of the discounted costs incurred over the lifetime of the system to the sum of the discounted actual energy values delivered. Rocha [26], Tervo [27] and Moschos [28] used this method for ESSs. Other methods used to evaluate ESS can be found in professional sources. These include Monte Carlo Simulation [2] and Real Options Theory [29]. However, to date, they have only been applied sporadically in the literature, compared with the abovementioned methods.

There is a wide range of methods for the economic evaluation of energy storage systems. The question is which method is suitable for the evaluating FESS technologies. FESS technologies represent a relatively new and innovative approach in the ESS field and are currently not commercially exploited to a great extent. This situation makes it difficult for stakeholders (e.g. energy system investors, contractors, and policymakers) to evaluate individual economic characteristics. Each system is unique and possesses several specificities. The investor must pay a significant amount for FESS technology to ensure a steady supply of electricity to the grid through energy storage. It was found that the FESS can be cost-effective when operated for at least 5,000 cycles per year with storage times of less than 60 minutes [30]. The cost associated with the use of FESS depends on several factors such as the type of use, the location of the specific technology, the construction method, the size of the investment, and the energy source used.

According to Abdon et al. [31] and Spataru et al. [32] finding an appropriate evaluation method to determine the cost-effectiveness of FESS technologies is difficult. The evaluation methods for conventional investment projects are inappropriate for this type of investment Rahman [10] and Zakeri [23] recommended the application of a method based on the life cycle cost (LCC) of a technology for its economic evaluation. This is because it allows for the consideration of the full life-cycle costs of the FESS, including manufacturing, installation, operation, maintenance, and disposal.

To apply the LCC method to a FESS, it is necessary to determine the total cost of manufacturing, installation, operation, maintenance, and disposal of the FESS. To determine the total capital costs associated with the FESS, the Total Capital Costs (TCC) method was used, which includes virtually all capital costs associated with the acquisition of the technology. Other factors to be included when using the LCC method for a FESS are the interest rate, investment lifetime, and inflation. These factors affect the total costs and subsequent returns and should be included in the calculations. The use of the LCC method for FESS also allows, where appropriate, comparison of different types of FESS and the determination of the best option for a particular application. This method allows the overall costs and benefits of the FESS to be considered and an informed decision (with certain assumptions) to be made as to whether the use of the FESS is economically viable.

## 3. Results

This study focused on the economic evaluation of energy storage in FESS. This relatively new technology is not widely used and is the subject of further scientific investigation.

We focused on two research questions:

Research Question 1: What is the most appropriate method for evaluating the economic efficiency of high-speed FESSs used to smooth out short-term fluctuations in the electricity transmission system?
Research Question 2: Can a high-speed FESS project cost-effectively compensate for short-term fluctuations in an electricity transmission system?

For this study, a high-speed FESS method using vacuum inside the flywheel enclosure was chosen, as proposed by a team of international researchers, and investigated in semi-operation. The purpose of this research was not only to discuss the issues of economic evaluation, but also to investigate whether modular high-speed FESS technology can be effective in the Czech environment (the prices of individual components and energy were determined by a survey of the Czech market and converted into euros). For this study, data obtained through collaboration in the international Power4Life project were used; the data were based on a study by Hlava et al. [8].

This is a high-speed FESS project potentially usable to compensate for short-term fluctuations in electricity transmission systems, with a total capacity of 25 modules of 1000 kWh, a planned frequency of one charge/recharge cycle per hour with continuous operation, and a lifetime of at least 20 years. The efficiency of this system is high (97%) owing to the incorporation of a flywheel into the vacuum housing, which reduces the loss of aerodynamic drag and improves the performance and safety of the system. The innovation potential of high-speed FESS can be seen in increasing the reliability of the power transmission system with the possibility of high absorption of renewable energy.

Based on expert research (see Literature review) and the experience of the research team, we developed an economic evaluation model based on the LCC method, discussed the suitability of using this method for project evaluation, analysed the risks associated with this method, and discussed possible scenarios resulting from the use of other evaluation methods.

An innovative life cycle cost analysis method was used to assess the economic efficiency of the project. Life-cycle costs represent the present value of the investment and operational recurring costs incurred throughout the lifetime of the system under study. Typically, these costs are expressed as a levelised annual cost, that is, they represent the amount that an investor would expect to pay annually for the entire operation of the energy storage system, including the repayment of the initial capital costs.

The LCCs of the module under study include levelised annual TCCs divided into three sub-items concerning the different expected lifetimes of the individual components (see Table 1), and also annual operation and maintenance costs.

**Table 1:** Total capital cost.

Cost item	Content of the item	Lifetime (years)	Price (in EUR)
C_PCS_	Inverter on the mains side, inverter on the storage side and inverter for active magnetic bearings	10	156 073
C_CSU_	Steel containment, active magnetic bearings, vacuum, hybrid rotor consisting of carbon fibre reinforced plastics (CFRP) and metal, electric drive unit (motor/generator),…	20	1 568 975
C_BOP_	Cost of project documentation, container, land rental, connection to the network, insurance,…	20	100 000

Source: expert estimate based on current market conditions (April 2023) and depending on the planned total capacity of 25 modules of 1000 kWh, a planned frequency of one charge/recharge cycle per hour with continuous operation.

The TCC method evaluates all the costs associated with the deployment of ESS technology. The TCC was calculated using Eq 1. It includes the costs of the power conversion system (PCS), costs associated with energy consumption and purchase, acquisition costs, cost for storage units (CSU)—costs related to energy storage (components of EnWheel modules), installation and delivery of the ESS unit, or cost of balance of the plant—BOP. The BOP includes costs for project engineering, grid connection interfaces, and integration facilities (e.g. transformers); construction management, including the cost of land and accessibility; other services and assets that are not included in the scope of PCS; and storage-related costs.


$$\text{TCC}=C_{PCS}+C_{CSU}+C_{BOP}$$ (1)

To calculate TCC correctly, Table 1 lists the individual cost items, useful lives, and expected acquisition costs.

The disposal cost at the end of the project was also a significant component of the C_BOP_ for battery storage. However, in the case of the FESS under consideration, these costs were not considered because, unlike battery energy storage, the disposal costs of inertial storage are negligible.

The levelised annual LCCs are then quantified according to Eq (2).


$$\text{LCC}=C_{PCS}\times CRF_{PCS}+C_{CSU}\times CRF_{CSU}+C_{BOP}\times CRF_{BOP}+C_{O\&\text{M}}$$ (2)

To calculate the levelised annual total capital cost, the time factor was considered through the capital recovery factor (CRF), as shown in Eq (3).

$$\text{CRF}=\frac{i\times {\left(1+i\right)}^{n}}{{\left(1+i\right)}^{n}-1}$$ (3)

where *n* is the lifetime in years, which varies according to the individual components of the FESS under consideration (Table 1); therefore, the CRF for each TCC item also varies. Furthermore, *i* is the interest rate considering inflation and other project risks, and the WACC (weighted cost of capital) model is used to estimate this interest rate. The WACC value represents the estimate of the minimum return required by an investor. In general, WACC can be expressed using Eq (4).

$$WACC=c_{D}\times D\times \left(1-\tau \right)+c_{E}\times E$$ (4)

Where *c*_*D*_ represents the cost of debt; *D* is the weight of debt; *c*_*E*_ is the cost of equity; *E* is the weight of equity and *τ* is the income tax. However, for the calculations in this study, the WACC value set directly by the Energy Regulatory Authority for the electricity sector in May 2022 [33] was used as the starting point.

Given that the ERO’s WACC of 5.3% is based on pre-2020 macroeconomic data, it may be considered unrealistic given the turbulent changes in the macroeconomic environment that occurred after 2020. The CNB’s base interest rate, which is significantly reflected in the cost of foreign capital, increased from 0.25% to 7.0% from 2020 to 2023 (as of 30 May 2023). The annual inflation rate increased from 3.2% to 15% by the end of 2019. The interest rate on long-term government bonds in the Czech Republic with a maturity of ten years, which is the risk-free basis for determining the cost of equity capital, increased from 2.13% to 7.21% between the end of 2019 and 31 December 2022 [34]. For this reason, the interest rate has been increased to 12% to consider the time factor in the CRF calculations and qualified estimates.

Annual operation and maintenance costs C_O&M_ were set at 2% of total capital costs in line with recommended practices for similar inertial storage facilities. For these technologies, available sources [35–37] report annual operation and maintenance costs between 1% and 3% of total capital costs.

The LCC sub-results are presented in Table 2.

**Table 2:** LCC calculation.

Levelised annual life cycle costs (LCC)–items	CRF	EUR/year
Levelised annual cost of CSU	0,1339	210 052
Levelised annual costs of PCS	0,1770	27 622
Levelised annual costs of BOP	0,1339	13 388
Annual operation and maintenance costs *C*_*O*&*M*_ (expert estimate)	X	5 021
**Life cycle costs (LCC)**		**256 084**

Source: own; data based on Table 1 using formula (2)

Under the above assumptions, the levelised annual LCC of EUR 256 084 represents the amount that the investor would expect to pay annually for the entire operation of the energy storage system, including the repayment of the initial capital costs.

At the planned average frequency of one charge (discharge) cycle per hour in continuous operation, 8760 cycles occur per year. The levelised annual **LCC** value **per cycle is then equal to EUR 29.23.** With a total output of 25 modules of 1000 kWh and a considered frequency of one cycle per hour, the annual **LCC** value **per MWh is EUR 29.23.**

It is quite common (e.g. [24]) to consider the cost of purchasing electricity from the grid as a factor affecting annual operation and maintenance costs in the LCC calculation. In our case, the cost of purchased electricity is not included in the LCC calculation because of the turbulent price development in the energy markets in the past and the principle of using the evaluated kinetic energy storage exclusively to compensate for fluctuations in the transmission system with a planned frequency of one charging/recharging cycle per hour in continuous operation. The cost of the purchased electricity is subsequently reduced by investors’ projected revenue from the sale of electricity. The amount of this potential margin (profit), quantified as the difference between the potential sale and purchase prices of electricity on an hourly basis, was compared with the LCC results to determine the economic efficiency of the investment. Owing to the procedure described above, using OTE intra-day market data, it is possible to use more accurate data for the calculation compared to the average annual electricity market prices. The values used for the calculation are the maximum margins that can be achieved on an hourly basis in the OTE Market (see S1 File).

The margin was adjusted to match the predicted efficiency of the proposed FESS (97%). This is because only 97% of the purchased and stored electricity can be sold in the market owing to the expected losses of electricity in storage, and the remaining 3% is the expected loss of electricity. The margin adjustment was as follows:

$$\text{adjusted}\;\text{intra-hour}\;\text{margin}\;\left(\text{profit}\right)=\text{maximum}\;\text{price}\times \eta –\text{minimum}\;\text{price}$$ (5)

Where η represents the efficiency of the system, which can be expressed by

$$\eta =\frac{E_{out}}{E_{in}}\;\left(\text{kWh}/\text{kWh}\right)$$ (6)

In which E_out_ and E_in_ are output and input electric energy.

The average adjusted margin is calculated based on the results of the intra-day OTE Market. This is the average difference of the adjusted intra-day price maxima and minima (on an hourly basis) for the period from 1 January 2022 to 30 June 2023.

**Fig 1:**
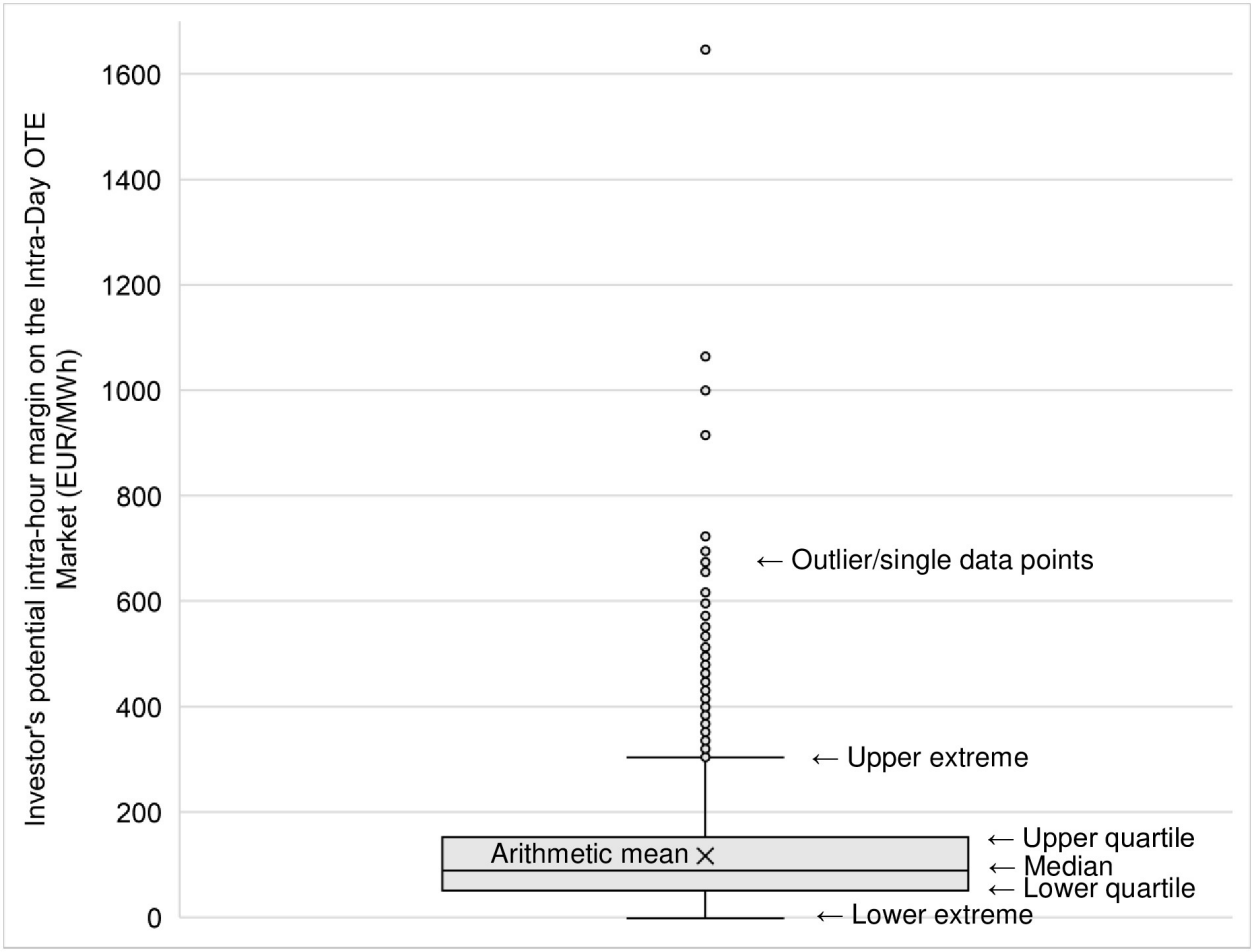
Investor’s potential intra-hour margin on the Intra-Day OTE Market (boxplot).

The arithmetic mean of the adjusted intra-hour margin in the OTE Market during the review was **116.39 EUR/MWh**. However, the average value of the difference between the adjusted maximum and minimum intra-day prices (hourly) in the OTE Market, expressed as an arithmetic average, can only provide an illusory indication of a project’s potential profitability. While the potential margin expressed as an arithmetic average is **EUR 116.39/MWh**, the dataset shows a standard deviation of EUR 97.19/MWh, indicating that the margin differed, on average, from EUR 116.39/MWh by EUR 97.19/MWh over the last year and half (see Table 3). Therefore, the degree of variability in intra-day prices over the previous year was high. The intra-day margin values range from EUR 4 to 1700/MWh (see Fig 1). If the median is used instead of the arithmetic mean, the cost reaches EUR 88.87/MWh. If the model uses the most frequently occurring value of the potential margin, it yields a value of EUR 94.00/MWh. In all cases of the median value of the potential margin, it should be noted that this is the maximum possible achievable intra-hour margin in the Intra-Day OTE Market.

**Table 3:** Indicators of the median value of the investor’s potential margin on the Intra-Day OTE Market for the period 1. 1. 2022–30. 6. 2023.

Mean value indicator	Indicator value EUR/MWh
Arithmetic mean^a^	116,39
Standard deviation	97,19
Median^b^	88,87
Mode^c^	94,00

Source: Own elaboration; data OTE [38].

^a^The arithmetic mean represents the average value of the intra-hour margin on the Intra-Day OTE Market calculated as the sum of all intra-hour margin values divided by their number.

^b^The median represents the middle value of the set of intra-hour margins on the Intra-Day OTE Market, sorted from the smallest value to the largest.

^c^The mode is the value that occurs most frequently in a given set (the most frequent value of the intra-hour margin on the Intra-Day OTE Market). The differences between the three mean values indicate an asymmetric frequency distribution of intra-hour margin values on the Intra-Day OTE Market.

Comparing the LCC per MWh of EUR 29.23 and the median possible margin realisable on the OTE intra-day market of EUR 88.87/MWh, it can be concluded that an FEES project using vacuum technology could be profitable, even assuming that the actual margin may not reach the median value. The final outcome also entails several risks related to the high degree of variability in predictions regarding the evolution of energy market legislation, the level of inflation, the associated increase in the input costs of investment, future developments in fiscal and monetary policy in individual economies, and other variables.

## 4. Discussion

The economic evaluation of ESS raises several questions. The methods by which the economic efficiency of individual facilities can be examined are appropriate for discussion, and the outcome of this process or the decision regarding the economic potential of a particular environmental investment is also important. Current knowledge suggests that ESS costs have recently been significantly reduced, and several economic analyses (e.g. [39]) have shown that these storage facilities are increasingly economically competitive with other energy sources such as fossil fuels.

In this study, an innovative life cycle costing (LCC) method was selected for the economic evaluation of an advanced FESS technology (using vacuum). Individual components were evaluated according to their price levels in the Czech Republic. As a result of the study, it was found that the designed technology operated under the postulated conditions can meet the efficiency requirements. It should be emphasised that this is a relatively new technology that has not yet been fully commercialised; therefore, it is necessary to work with certain assumptions and limitations. The risk factors in the energy market and inflationary pressures on the Czech market are not entirely clear, which may affect the prices of individual components. The parameters assumed in a technical solution can also be problematic. The technology is new, and there are insufficient reference data available to allow for the correction of the technical solution.

Several authors (e.g. Moschos [28], Tervo [27] among others) have recommended the LCOE (LCOS) method, which is more flexible because it allows the comparison of technology costs in different system designs and operating modes. This can be misleading in situations where the amount of electricity supplied by the system in each year of its lifetime is not precisely known (somehow similar to Tervo [27]).

In the research section, the methods used to evaluate conventional commercial investments (e.g. NPV or IRR) are discussed. These models focus only on the monetary aspect of investment evaluation, and work with a relatively clearly defined risk [40]. However, ESS systems currently exhibit several uncertainties, not only in the technology area (efficiency, durability), but also in the economic area (amount of capital or operating costs), and thus the usefulness of these methods is significantly reduced.

In this study, based on the research conducted, the LCC method was selected as the most appropriate method for evaluating the economic efficiency of a high-speed FESS used to compensate for short-term fluctuations in the power transmission system (Research Question 1) but was modified. Several authors have recommended the LCC method because of its ability to assess the total cost of machinery and equipment acquisition, defining the present value of all capital and recurring costs incurred over the lifetime of the system. From this point of view, LCC is the most important model for evaluating and comparing different EES systems [23, 41]. The LCC quantification is based on TCC, which can be somewhat problematic since EES technologies have not been used in practice for a long time and there is not enough experience with the replacement of individual components during their life cycle and the associated costs (risk of price increase in the long term, pricing policy of suppliers, etc.) [23]. In addition, the use of the flywheel storage is specific and consists of repeated short-term use to compensate for fluctuations in the electricity transmission system. For these reasons, the cost of power purchase was excluded from the calculation of the total project cost. These costs were considered only taken into account when calculating the margin.

The LCCs per MWh were compared with the average intra-hour margin realisable in the OTE Market, and the efficiency of the flywheel storage was also considered in the margin calculation. Under the modelled technical and economic conditions, it was found that a high-speed FESS project that can compensate for short-term fluctuations in the electricity transmission system can be economically efficient in the Czech Republic (Research Question 2).

In summary, the economic evaluation of electricity storage systems is subject to uncertainties. Among the important risk factors that need to be reflected are in particular the development of legislative regulation of the energy market, the level of inflation and the associated increase in input costs for investments, future developments in the fiscal and monetary policy of individual economies and, last but not least, the uncertain geopolitical situation and the related lack of a good prediction of the development of electricity prices in the longer term.

## 5. Conclusion

ESS are currently one of the most studied elements of modern power systems. The shift from fossil fuels towards renewable energy sources has great environmental benefits for society but brings new challenges in terms of storing energy so that it is available on demand. There are many ESS systems available for this purpose. Batteries are one of the most widely used types of batteries. The efficiency of battery storage systems has improved significantly over the years, and the cost of production has decreased. This makes battery storage increasingly competitive with traditional energy sources such as fossil fuels. However, the use of batteries in ESS poses environmental burdens and risks. Research on new materials and technologies that enable renewable energy storage has become increasingly important. One method involves functionally integrated magnetic flywheel storage in a vacuum environment (FESS). This is a relatively new method that has not yet been widely commercialised, and both technical and economic issues are associated with its use.

Professional sources offer a range of methods for the economic evaluation of projects. However, some methods are unsuitable for evaluating the economic efficiency of investments in the environmental field. The economic evaluation of energy storage involves analysing the costs and benefits of a given project to assess its economic efficiency in a broader context. Thus, the technical parameters of the proposed project, such as the performance, efficiency, and lifetime of the systems, and their cost of routine operation and possible maintenance, are also important factors for economic assessment.

The research activities were directed towards an economic assessment of the FESS technology using a vacuum environment for its operation. Based on the sources studied, an innovative LCC (the life cycle cost) method was selected for evaluation. This method calculates the total project cost over the lifetime of the project. The valuation of the individual components of technology and energy is based on the price level in the Czech Republic. The cost of technology acquisition was calculated using the Total Capital Cost (TTC) method and included all significant sub-components. The operation and maintenance costs were included, but not the cost of purchasing electricity from the grid; these costs were considered in the margin calculation. The LCC per MWh was compared to the average intra-hour margin realisable in the OTE Market, and the efficiency of the flywheel storage was also considered in the margin calculation. Under the modelled technical and economic conditions (lifetime and performance), it was found that investment could be evaluated as efficient under the conditions of the Czech Republic. However, there are always assumptions and limitations to be discussed, which need to be addressed when evaluating these new technologies, alongside the relevance of using other methods of economic evaluation.

The selection of an appropriate type of energy storage system depends upon many parameters, and it is important to choose a system with an optimal cost-to-performance ratio that can meet the technical requirements of a specific task. Other factors also play important roles and must be considered when deciding on a particular system or its economic viability. These include legislative aspects, geopolitical situations, inflation forecasts, and a country’s fiscal or monetary policies. ESS, and especially FESS, represent interesting and innovative technologies with great technical and economic potential for specific applications. It can be assumed that with their commercial dissemination, sufficient technical and economic data will be available to make a fully informed assessment of the technical and economic efficiency of individual technologies.

## Supporting information

S1 File OTE Intra-day Market results. (XLS)
